# A Quantum Chemistry Approach Based on the Analogy with π-System in Polymers for a Rapid Estimation of the Resonance Wavelength of Nanoparticle Systems

**DOI:** 10.3390/nano9070929

**Published:** 2019-06-28

**Authors:** Alessandro De Giacomo, Zita Salajkova, Marcella Dell’Aglio

**Affiliations:** 1 Department of Chemistry, University of Bari, Via Orabona 4, 70126 Bari, Italy; 2 CNR-NANOTEC c/o Chemistry Department, University of Bari, Via Orabona 4, 70126 Bari, Italy; 3 Central European Institute of Technology (CEITEC), Brno University of Technology, Purkyňova 656/123, 612 00 Brno, Czech Republic

**Keywords:** plasmon, nanoparticle, NPs coupling, surface interaction

## Abstract

In this paper, the Variational Method based on the Hückel Theory is applied to NPs chain and aggregate systems in order to estimate the energy of the plasmon and, in turn, the resonance wavelength shift, which is caused by the interaction of adjacent NPs. This method is based on the analogies of NPs dipole interactions and the π-system in molecules. Differently from the Hartree-Fock method that is a self-consistent model, in this approach, the input data that this method requires is the dimer energy shift with respect to single NPs. This enables us to acquire a simultaneous estimation of the wavefunctions of the NPs system as well as the expectation energy value of every kind of NPs system. The main advantage of this approach is the rapid response and ease of application to every kind of geometries and spacing from the linear chain to clusters, without the necessity of a time-consuming calculation. The results obtained with this model are closely aligned to related literature and open the way to further development of this methodology for investigating other properties of NPs systems.

## 1. Introduction

The understanding of light-matter interaction at a nanoscale has been developed rapidly in recent years. Various models [1,2,3] have been proposed to understand and optimize several applications such as Surface Enhanced Raman Scattering (SERS) [4], photo-emission [5], fluorescence [6], Nanoparticle Enhanced Laser Induced Breakdown Spectroscopy (NELIBS) [7], and more. It is well known that, when an incident radiation illuminates a metal nanoparticle (NP), it induces polarization of the NPs conduction electrons and, if the radiation frequency is in resonance with the plasmonic frequency of the particle itself, a strong electromagnetic field is induced on the border of the NPs [8]. If the radiation with adequate frequency interacts with a set of NPs placed at a distance, which is smaller than the diameter of the NP, a strong field enhancement is generated in the gap between the NPs, i.e., the so-called “hot spot” [9,10]. In this case, an effective variation of the Coulomb restoring force that exists between the positive metallic lattice of the NPs and the oscillating electrons occurs because of the interaction of adjacent NPs dipoles. This results in a notable change of the system’s resonance energy. Several experiments and models have been performed in order to quantify this phenomenon and, thus, determine the resonance wavelength of the NPs system [11,12]. Although various compositions of multi-NPs have been investigated [13,14], due to the mathematical approach complexity and the difficult experimental realization of the controlled structure at the nanoscale level, most studies have focused on the dimeric system of NPs. In this frame, the nature and the dependence on the particle size, as well as on interparticle distances, in the dimeric system, has been largely investigated and numerous articles have been published [11,15] outlining a full description of NPs dimer both from theoretical and experimental point of views. The classical approach for studying plasmon effects is based on the determination of the permittivity function. This is done by modeling the optical properties of the metals using different multi-parameter methods. Even though this classical approach is very useful, it becomes less sufficiently accurate and extremely time consuming when applied in investigating a complex system of NPs. On the other hand, NP interactions can be studied in analogy with atom interactions during the formation of molecules [16,17]. In this frame, an individual NP can be described with a wavefunction and their interaction can be studied in the same way as the interaction of atomic orbitals in the Hückel theory. Good examples are the case of π-bonding in the dienic system and the molecular orbital interactions between ligands and metal in coordination chemistry. This second approach enables us to apply the methodologies used to determine molecular bonding and the energy changes during the atomic orbitals interaction.

In this paper, we propose a quantum mechanics approach, called the Hückel’s Variational Method (VM) [18], inspired by the analogy between NP systems and the molecular orbital theory, which was initially proposed by Nordlander at al. in the hybridization model [16,19], for a rapid and simple estimation of energy and the resonance wavelength in the NPs chain and cluster systems. This approach requires input data that has been already reported in literature i.e., the energy shift of simple systems such as dimers or single NPs on the surface [15,20]. They enable us to obtain information on more complex NPs systems without having to resort to time consuming calculations. Apart from self-consistent methodologies [11] for multiple metal NPs where the random phase approximation in the secular determinants is also used to estimate the surface plasmon resonant energies that, in turn, depend on the nanoparticle spacing, this approach requires the energy shift of simple systems as initial input data, such as dimers and single NPs on the surface that has already been reported in literature [15,20]. These input data of the energy, which contains the dependence on the nanoparticle distance and surrounding medium, enable us to obtain information on more complex NPs systems without having to resort to time-consuming calculations. Beyond the practical result, i.e., the determination of the resonance energy shift, the investigation of NPs system with the VM provides an insight of the analogies and differences between NPs plasmon and the π-molecular orbital.

## 2. The Variational Method Applied to NPs Systems

The Variational Method is the most widely used approach for the calculation of energy and π-orbitals in molecular chemistry, such as Hückel theory. The best wavefunction Ψ representing the system of elements and consisting in a linear combination of the wavefunction representing each single element is the one that minimizes the expectation value of the energy. In this frame, a function of the energy depending on the weight coefficients of the linear combination is obtained. The minimum value of this function E = f(c1, c2, c3,…cn) is greater than or equal to the exact value of the energy. In this light, finding the minimum of this function with respect to the weight coefficients allows the determination of the closest value to the exact energy of the system [18]. In this case, as suggested in Reference [17], the collective character of the plasmon is described in terms of the linear combination of single-particle excitations and, therefore, a time-dependent model can be bypassed, in the approximation with the use of empirical input data. Although the same method can be applied to other physical quantities, in this instance, we will apply it for the energy of the system, considering that we are focused on the determination of the resonance wavelength, that is linked to the energy by the relation E = hc/λ, where E = ω is the energy of the plasmonic system.

Let us consider a general wavefunction of a linear system of n equally sized and spaced NPs given by the following linear combination.

(1) $$\psi_{k}=c_{1}\varphi_{1}+c_{2}\varphi_{2}+c_{3}\varphi_{3}+\ldots +c_{n}\varphi_{n}$$

where *φ* is the wavefunction associated with the dipole oscillation of an excited individual NP.

If we use n different basis for the linear combination, n different wavefunctions will be obtained, with each one describing a different mode. In this case, we will consider only longitudinal modes, restricting our attention to those modes which are susceptible when the incident radiation is perpendicular to the NPs cluster.

The expectation value of the energy is given from the following equation, where $\hat{\omega }$ is the Hamiltonian operator.

(2) $$\omega =\frac{\int_{\tau }\psi \hat{\omega }\psi d\tau }{\int_{\tau }\psi \psi d\tau }=\frac{\int_{\tau }(c_{1}\varphi_{1}+c_{2}\varphi_{2}+c_{3}\varphi_{3}+\ldots +c_{n}\varphi_{n})\hat{\omega }(c_{1}\varphi_{1}+c_{2}\varphi_{2}+c_{3}\varphi_{3}+\ldots +c_{n}\varphi_{n})d\tau }{\int_{\tau }(c_{1}\varphi_{1}+c_{2}\varphi_{2}+c_{3}\varphi_{3}+\ldots +c_{n}\varphi_{n})(c_{1}\varphi_{1}+c_{2}\varphi_{2}+c_{3}\varphi_{3}+\ldots +c_{n}\varphi_{n})d\tau }$$

Developing the integral of Equation (2) and setting for each value of *j* with 1 ≤ *j* ≤ *n*, the following derivative equals zero.

(3) $${\left(\frac{\partial \omega }{\partial c_{j}}\right)}_{c_{1},c_{2},c_{3}\ldots c_{n}}=0$$

We obtain the following system of secular equations.

(4) $$\left|\begin{array}{ccccc} \left(\Omega_{11}-\omega \right) & \left(\Omega_{12}-S_{12}\omega \right) & \left(\Omega_{13}-S_{13}\omega \right) & \left(\Omega_{14}-S_{14}\omega \right) & \ldots \\ \left(\Omega_{21}-S_{21}\omega \right) & \left(\Omega_{22}-\omega \right) & \left(\Omega_{23}-S_{23}\omega \right) & \left(\Omega_{24}-S_{24}\omega \right) & \ldots \\ \left(\Omega_{31}-S_{31}\omega \right) & \left(\Omega_{32}-S_{32}\omega \right) & \left(\Omega_{33}-\omega \right) & \left(\Omega_{34}-S_{34}\omega \right) & \ldots \\ \ldots & \ldots & \ldots & \ldots & \ldots \\ \left(\Omega_{n1}-S_{n1}\omega \right) & \left(\Omega_{n2}-S_{n2}\omega \right) & \left(\Omega_{n3}-S_{n3}\omega \right) & \ldots & \left(\Omega_{nn-1}-S_{nn-1}\omega \right) \end{array}\begin{array}{c} \left(\Omega_{1n}-S_{1n}\omega \right)\\ \left(\Omega_{2n}-S_{2n}\omega \right)\\ \left(\Omega_{3n}-S_{3n}\omega \right)\\ \ldots \\ \left(\Omega_{nn}-\omega \right) \end{array}\right|•\left|\begin{array}{c} c_{1}\\ c_{2}\\ c_{3}\\ \ldots \\ c_{n} \end{array}\right|=0$$

where

(a) the Coulomb’s integral

$\Omega_{ii}=\int_{\tau }\varphi_{i}\hat{\omega }\varphi_{i}d\tau =\omega_{0}$ that is the energy of the single particle,

(b) the interaction integrals

$\Omega_{ij}=\Omega_{ji}=\int_{\tau }\varphi_{i}\hat{\omega }\varphi_{j}d\tau =\int_{\tau }\varphi_{j}\hat{\omega }\varphi_{i}d\tau =\beta \approx -\Delta \omega_{\dim}$ is equal to the shift of energy of the dimer compared to the single NP when i and j represent adjacent NPs and is null for non-adjacent particles because we assume that non-adjacent dipoles do not interact between them. In the case of a linear chain of NPs, Ω*_ij_* = *β* when |i−j|=1 and is equal to 0 when |i−j|>1;

(c) the overlapping integral

$S_{ij}=S_{ji}=\int_{\tau }\varphi_{i}\varphi_{j}d\tau =\int_{\tau }\varphi_{j}\varphi_{i}d\tau \approx 0$ because the NPs are far enough to neglect the contribution of the efficient wavefunctions overlapping. 

Under these conditions, for the system of equation to be null, the secular determinant must be numerically equal to 0. As an example, in the case of a linear chain of n NPs, the following condition must be assumed.

(5) $$\left|\begin{array}{ccccc} \left(\omega_{0}-\omega \right) & \left(\beta \right) & 0 & 0 & \ldots \\ \left(\beta \right) & \left(\omega_{0}-\omega \right) & \left(\beta \right) & 0 & \ldots \\ 0 & \left(\beta \right) & \left(\omega_{0}-\omega \right) & \left(\beta \right) & \ldots \\ \ldots & \ldots & \ldots & \ldots & \ldots \\ 0 & 0 & 0 & \ldots & \left(\beta \right) \end{array}\begin{array}{c} 0\\ 0\\ 0\\ \ldots \\ \left(\omega_{0}-\omega \right) \end{array}\right|=0$$

Some other examples are reported in the supplementary materials Figure S1. The solution of this determinant for n particles, i.e., n wavefunctions, gives n energy modes, including some that are bright modes (ω < ω_0_) while the rest are dark modes (ω > ω_0_).

**Figure 1:**
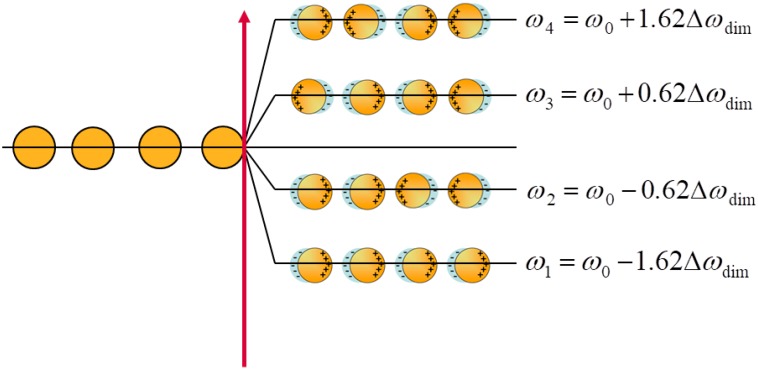
Interaction between 4 NPs in the chain resulting in four different modes.

A representative sketch in the case of a chain of four equal NPs is shown in Figure 1.

As a result of this calculation, we obtain several wavefunctions describing several dipole configurations, as shown in the example reported in Figure 1.

It is worth underlining that, with the present approach, whenever the plasmon energy of the single NP and the shift of energy of the dimer with respect to the single NP is known, the calculation of the secular determinant of Equation (5) is a minor and immediate operation. Thus, this method shows a general trend of the NPs system where the effect of NPs distances is included in the interaction integral whose value can be obtained by using an independent theoretical or experimental approach. The exact wavefunction of the single NPs is not required for the computation of the energy in agreement with the Hückel theory and so the method can be applied virtually to all the geometries of the single element as the effect of the properties of the single NPs in the complex system, which is contained in the input data ω_0_ and Δω_dim_. As is well known, dielectric polarization plays an important role in the plasmon resonant energy. For this reason, the input data should be selected properly, taking into account the dielectric medium where the NPs system is embedded.

Dimeric systems have been intensively studied in the last decade because the optical properties of this kind of system can be determined accurately using different experimental and theoretical approaches [21,22,23,24,25,26,27,28,29,30]. For instance, we reported in Table 1 some data retrieved from published works on Au and Ag NPs dimers.

## 3. NPs Chain Systems

**Figure 2:**
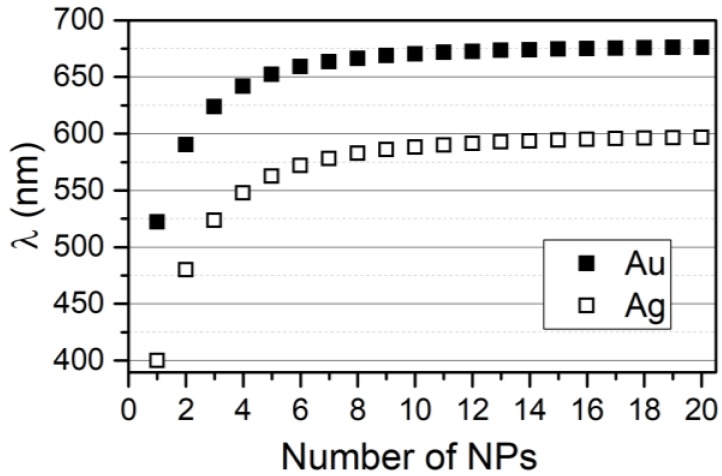
Lowest energy mode of the chain as a function of the number of NPs, for gold and silver NPs. Input data for AuNPs and AgNPs is, respectively,ω_0_ = 2.375 eV, β = −0.28 eV, ω_0_ = 3.10 eV, and β = −0.52 eV.

Let us consider the case of a chain of AuNPs and one of AgNPs, where the plasmon energy ω_0_ and the dimer shift Δω_dim_ are ω_0_ = 2.375 eV, β = −0.28 eV and ω_0_ = 3.10 eV and β = −0.52 eV, respectively, for AuNPs of 40 nm and AgNPs of 50 nm [22]. Results of the VM are reported in Figure 2, which shows an increase of the wavelength as a function of the number of NPs in the chain, which, in turn, results in the red-shift of the resonance wavelength of the lowest energy mode, obtained with the solution of the secular determinant of Equation (5).

As expected, the wavelength of the longitudinal modes shifts to higher values as the number of NPs in the system increases. This moves toward a plateau for n → ∞. Although, with the present model, we are not investigating the behavior of the Hot Spot [31,32] generated in the NPs system. However, it is possible to estimate just the number of Hot Spots assuming they are equal to the number of coherent interactions between adjacent NPs. 

For each mode, the number of available coherent dipole interactions, i.e., potential hot spots, is m = n–k, where n is the number of NPs constituting the system and k is the mode number, assuming k = 1 for the lowest energy mode. This observation makes it clear that the maximum number of available coherent dipole interactions is obtained for the mode with the lowest energy. This mode represents the dipole configuration, which allows the maximum electromagnetic field enhancement. On the contrary, the number of sites where the dipole assumes the opposite orientation corresponds to the number N of nodes of the wavefunction representing the specific mode, so that N = n − 1 − m = k − 1. Consequently, in order to polarize a given mode, it is necessary that the number of constructive interactions between the dipoles is greater than the number of dipoles with a destructive interaction. This means that the number of coherent dipole interactions has to be greater than the number of nodes, m > N (see Figure 1). This observation suggests that, in a bright mode m > N, consequently, ω < ω_0,_ and the corresponding wavefunction represents a system with a net total dipole. On the contrary, when m < N, then ω > ω_0_, and the wavefunction describes a system where no effective dipole exists, which is a dark mode [33]. In the case of the dark mode, the total wavefunction presents more nodes than coherent interactions and, therefore, in agreement with the general knowledge, they cannot be excited.

**Figure 3:**
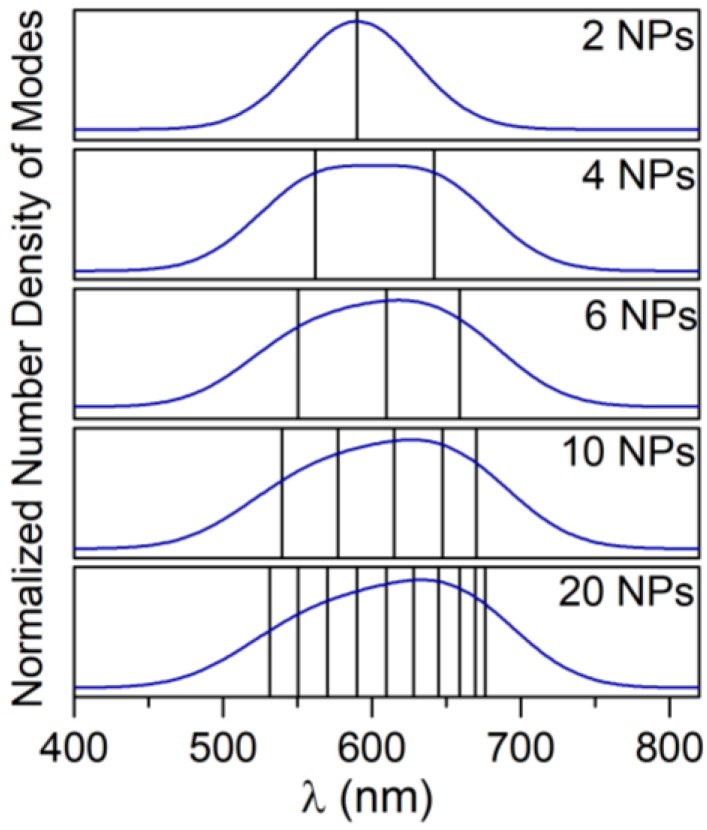
The image of the normalized number density of modes for chains with five different lengths. Black vertical lines represent single modes as obtained with the Variational Method, blue curves represent the density of states, calculated as a convolution of gauss curves modeled for each mode using a broadening of 80 nm for each state.

Considering the broadening of each state due to the quantum limit of the investigated system [34], it should be expected that all these states merge together in a wide band. Consequently, the system of NPs can be suitably polarized along a broad range of incident electromagnetic field frequencies generating a band of resonance including all the bright modes within the limits of possible configurations, which the dipoles in the chain can assume. Since the VM does not allow taking into account the broadening of the energy levels resulting from the quantum limits and a detailed investigation on the broadening would be required [35,36], with the only aim to show how the broadening of the states may form a band of resonance, in Figure 3, we also report the convolution of the modes, assuming a Gaussian shape with a broadening (FWHM, Full Width at Half Maximum) of 80 nm in agreement with the broadening of the SPR (Surface Plasmon Resonance) of the single AuNPs of 40 nm [34]. Even though, this is an arbitrary condition for a chain system and it underestimates the real broadening, it gives an idea of the correlation between the states of the system and the peculiarities of the band of resonance generally observed in the experimental applications.

This phenomenon underlines the most important feature of the NPs systems: the possibility to adapt the configuration of their dipoles to the incident electromagnetic radiation, which allows a sort of cooperation in reaching resonance with the incoming perturbation. Clearly, this effect is not achievable with more rigid systems, e.g., the atomic ones, where resonance occurs only at a specific frequency. This flexibility of the NPs systems in reaching resonance with the perturbation occurs as an effect of a system that borders strongly quantized systems such as atoms and macroscopic (i.e., not quantized) systems such as particles.

**Figure 4:**
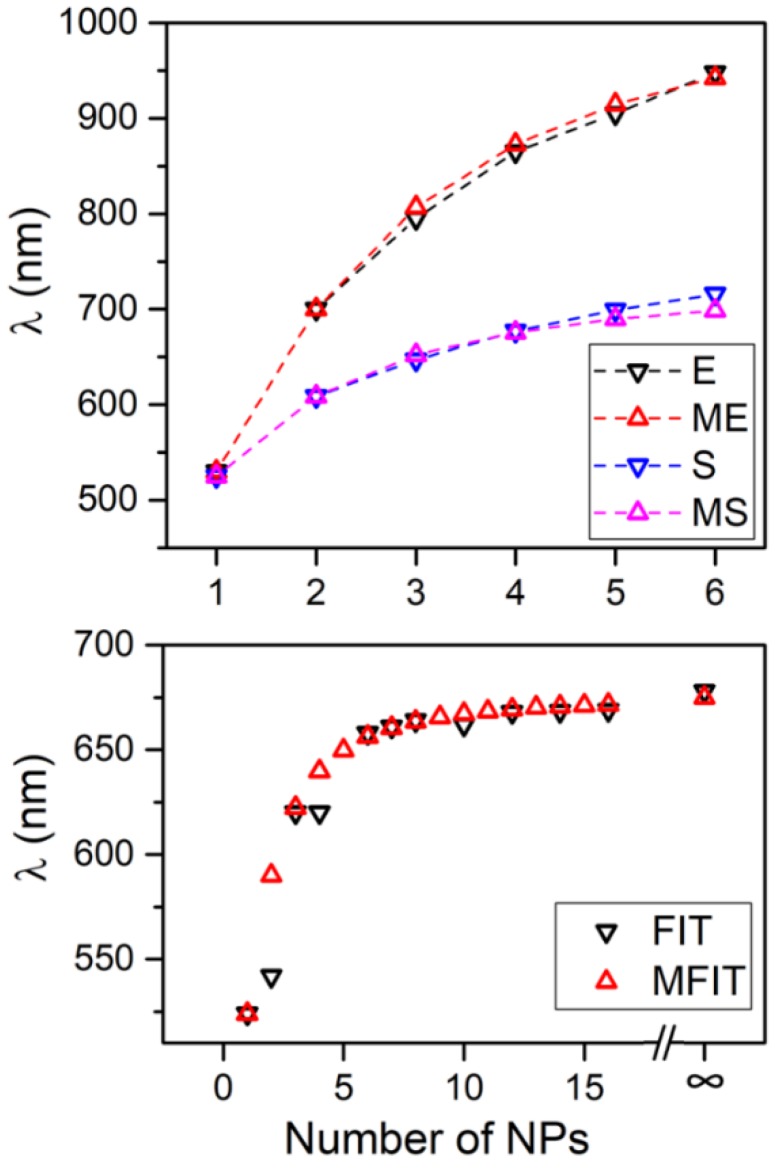
Comparison of results obtained by the variational method with data presented in literature. E (Experimental), S (electrodynamic Simulation), and FIT (Finit Integration Technique) have been taken from References [22,24,26], respectively. ME (ω_0_ = 2.34 eV, β = −0.57 eV), MS (ω_0_ = 2.36 eV, β = −0.33 eV), and MFIT (ω_0_ = 2.37 eV, β = −0.27 eV) are the results calculated by the Variational Method for the appropriate systems.

It is interesting to compare the results of the VM with other results based on different approaches reported in literature, as shown in Figure 4. In this case, we compare the lowest energy mode obtained with VM with different properties reported in literature and related to the resonance energy of the plasmonic system. Observation of Figure 4 indicates an impressively high level of agreement of the results of VM with those obtained with Electrodynamic Simulation, S, [22] and Finit Integration Technique, FIT, [26], as well as with experimental results, E, [24]. The values of ω_0_ and β used for comparisons of Figure 4 are ω_0_ = 2.34 eV, β = −0.57 eV for the comparison with experimental results (EM), ω_0_ = 2.36 eV, β = −0.33 eV for the comparison with Electrodynamics simulation (MS) and ω_0_ = 2.37 eV, β = −0.27 eV for the comparison with the Finit Integration Technique (MFIT). The agreement of the proposed method with literature data shows the similarity of the plasmonic characteristics of NPs system with a conjugated dienic system, where this theoretical approach is considered one of the most accurate ways to determine the energy of π-bonding. 

## 4. NPs Cluster Systems

**Figure 5:**
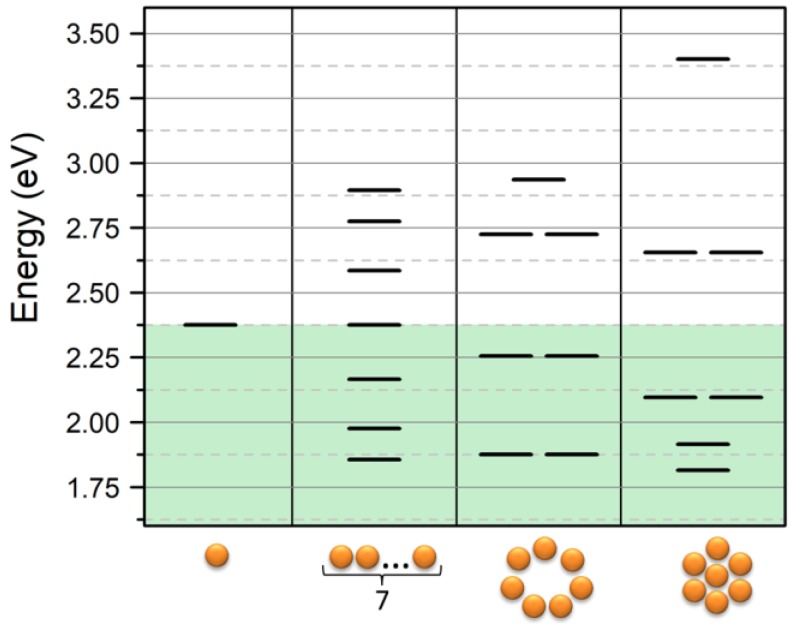
Energy diagram of the linear combination obtained in the case of heptamer geometries: linear, ring, and cluster. Input data are ω_0_ = 2.375 eV and β = −0.28 eV.

One of the most attracting results that can be obtained with the VM is the determination of resonance energy of a more complex system than a dimmer and, as mentioned in the introduction, the VM allows us to study the interaction between NPs in a different geometrical configuration. As an example, let us consider the heptamer in three geometries: linear chain, ring chain, and cluster. The energy diagram obtained for these systems is reported in Figure 5. Same as in the previous section, we considered a system of Au-NPs with ω_0_ = 2.375 eV and β = −0.28 eV. We can note that the linear chain and ring chain had a similar energy shift, 1.86 eV and 1.88 eV, respectively. However, while in the linear chain, there was no degeneracy of the modes, the ring chain presented a degeneracy equal to 2 of all the modes except the one with the higher energy with k = 7. This effect was due to the geometrical symmetry of the ring system. When comparing the chain systems with the heptamer cluster, a further redshift was observed as a result of the strong coupling with the NP placed in the center in the heptamer cluster. It interacts with all the surrounding NPs, which increases the number of coherent interactions. The latter observation introduces a general feature of 2D systems, which is an increase of the number of bright modes with respect to the dark modes. In this frame, the heptamer shows a general condition where the NPs packing (i.e., the ratio between NP diameter and interparticle mean distances) is optimized, which allows the central NP to have a full interaction with several other units, since it will be discussed below in the section about the 2D lattice of NPs.

**Figure 6:**
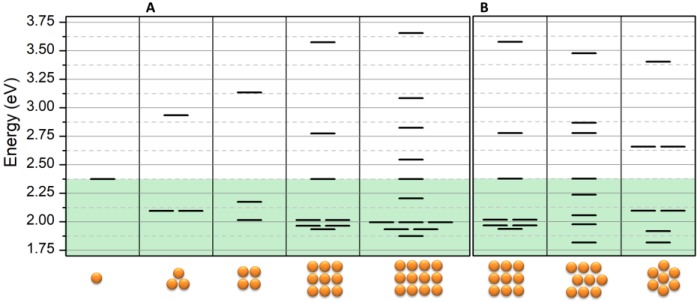
Energy diagram of the linear combination obtained in the case of 2D geometries: (**A**) effect of increasing the number of NPs in the cluster on mode formation, (**B**) effect of packing on mode energy. Input data are ω_0_ = 2.375 eV and β = −0.28 eV.

As a second case, we investigated the effect of ordered 2D NPs clusters. It was necessary to take into account that there were different types of interaction between adjacent NPs and particles placed on the diagonal direction. Therefore, for the latter ones, we used a different value of the interaction integral, which is β′ = β/√2, according to geometric considerations (see supporting information). As a result of the dipole’s interaction, a new energy diagram was obtained and is reported in Figure 6A. The figure shows that the energy of bright modes decreases with the increasing of NPs in the reticular cluster and that the number of bright modes is always greater than the one of dark modes. As mentioned above, this is due to the increasing number of coherent dipole interactions. For this reason, and because the total energy of the levels must be conserved, the dark modes notably increase their energy. Once again, for ω < ω_0_, the energy stabilization and, in turn, the wavelength increasing is dependent on the number of interactions that every NP is able to perform.

The ordered reticular system of NPs does not represent the best situation since the packing factor can be improved by decreasing the average distances between the NPs. This effect is demonstrated in Figure 6B where the energy diagram of the 2D lattice is reported. The figure clearly shows a further decrease of the energy of about 0.1 eV corresponding in a wavelength red-shift of about 40 nm when the chain composing the 2D-lattice is shifted with respect to the adjacent chains in order to optimize the packing of the NPs.

In Table 2, for a fast comparison of resonance energy of different cluster geometries, the energy and the corresponding wavelength of the lowest energy mode (k = 1), that, as mentioned above, corresponds to the main peak of the resonance band, is reported at ω_0_ = 2.375 eV, β = −0.28 eV.

**Figure 7:**
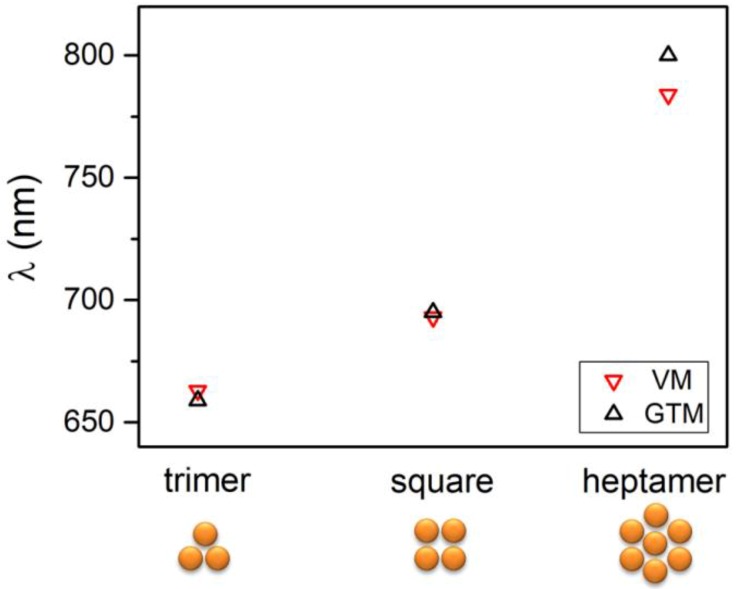
Comparison of results of the present work and Reference [37] in the case of triangle, square, and heptamer clusters. Input data have been estimated from Reference [37] and are ω_0_ = 2.15 eV and β = −0.28 eV.

Lastly, in Figure 7, we compared the case of triangular, square, and heptamer cluster with some results published in Reference [37]. For all the examined structures, there is good agreement of the resonance wavelength obtained with Generalized Mie Theory (GMT) and the wavelength corresponding to the lowest energy mode, as calculated applying the VM.

## 5. Effect of the Surface

**Figure 8:**
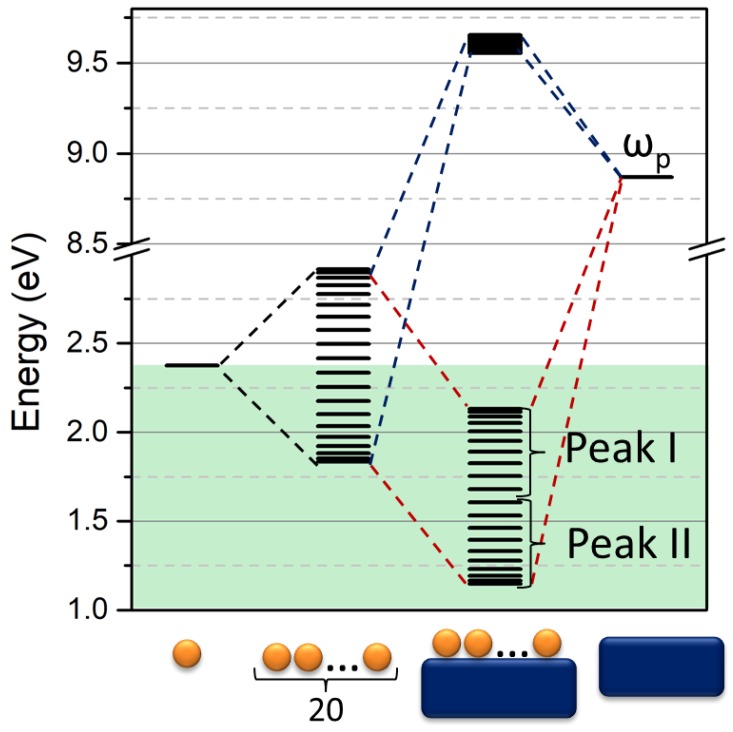
Sketch of the linear combination in the Variational Method for a 20 NPS chain on a gold surface. Black lines symbolize modes of three different systems (from left to right): 20 NPs chain, 20 NPs chain on the surface, and the surface with the plasmon energy ω_p_ = 8.87 eV. The green region illustrates the position of the bright mode ω < ω_0_ with ω_0_ = 2.38 eV.

The effect of the metallic surface on the chain plasmon resonance is of great interest and has been investigated by several authors (see [20,38,39,40,41]). Two approaches may be feasible in the context of the VM. Applying the first approach, we can directly couple the surface plasmon energy independently with all the single NPs constituting the chain. From the mathematical point of view, it means adding a further equation in the system as well as adding a second term, α, that takes into account the energy shift due to the interaction of an individual NP with the metallic surface. In any case, this approach would take into account only the main energy shift due to a strong coupling of the metal surface and the NPs. On the contrary, the surface can interact with the NPs chain, which induces two main groups of modes, with one representing the most effective coupling and the other one’s surface interacting destructively with the modes of the chain [20]. In order to better represent this phenomenon, we propose coupling directly each chain wavefunction with the metallic surface. Consequently, each single mode determined, as discussed in the previous paragraph, is coupled with the metal plasma energy of the surface where the chain or cluster is placed. This obtains a 2 × 2 matrix for each mode. Thus, the determinant becomes:

(6) $$\left|\begin{array}{cc} \left(\omega_{k}-\omega \right) & \alpha \\ \alpha & \left(\omega_{S}-\omega \right) \end{array}\right|=0$$

where ω_k_ is the resonance energy of the mode k of the chain, ω_S_ is the plasma energy of the metal, and the interaction integral α is the shift in energy due to the interaction of the chain with the surface. A sketch of the level formation is shown in Figure 8 for the chain system and for the chain on the metallic surface.

**Figure 9:**
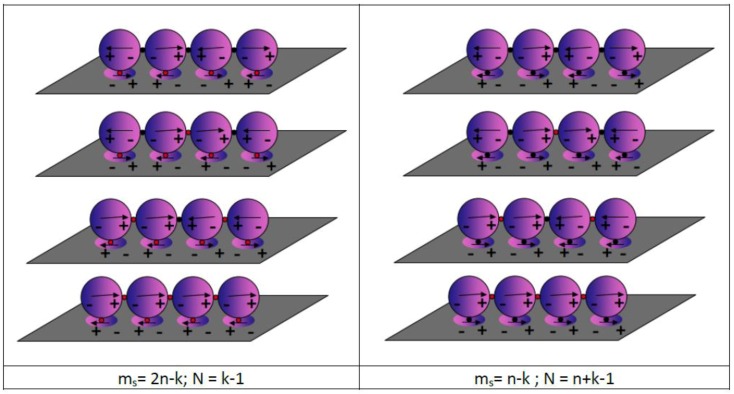
Dipole orientation during four NPs chain on a metallic surface in the case of (**left**) bright and (**right**) dark modes. Potential hot spots are indicated with red spots and nodes are indicated with black spots.

For large numbers of NPs (N > 10) in the chain, the interaction with the surface generates three groups of levels. The first one concerns the influence of NPs system on a surface where the energy is beyond the plasma energy of the metal. It is clearly a set of dark modes. The other two groups represent two sets of modes of the chain on the metallic surface, where the interaction of the surface with the chain decreases the energy of both low and high energy modes of the insulated chain. Moreover, it is worth underlining that the dark modes of the insulated chain become bright under the effect of interaction with the surface. It clearly explains the nature of the second peak when a chain of NPs is placed on the metallic surface [39,40]. This issue is very interesting as the introduction of the surface in the investigated system allows n new coherent dipole interactions due to the interaction of every single particle constituting the chain and the surface plasmon. In this view, the number of coherent dipole interactions where hot spots can be formed becomes m_s_ = 2n − k for the positive interaction with the surface while the number of nodes remains the same as the insulated chain. On the contrary, for the destructive interaction of the chain with the surface, the number of nodes becomes N_s_ = n + k − 1, while the number of coherent dipole interactions remains the same as the insulated chain. This effect is shown in Figure 9 in the case of a four NPs chain.

In order to explore the potentiality of this method, we calculated the resonance energy of the modes related to a chain of gold NPs on a gold surface, assuming the input data as follows: ω_s_ = 8.9 eV, α = −2.6 eV, with ω_k_ calculated using the VM, as described in the previous paragraph with ω_0_ = 2.38 and β = −0.28 eV. By a formal point of view, it would be more correct to use the energy of the metal surface plasmon (6.4 eV) and the corresponding interaction integral, but, as from the practical point of view, the energy of the metal is only the baseline for the calculation. We decided to use the plasma frequency (8.9 eV) and the corresponding interaction integral, which is more easy to estimate from literature data.

**Figure 10:**
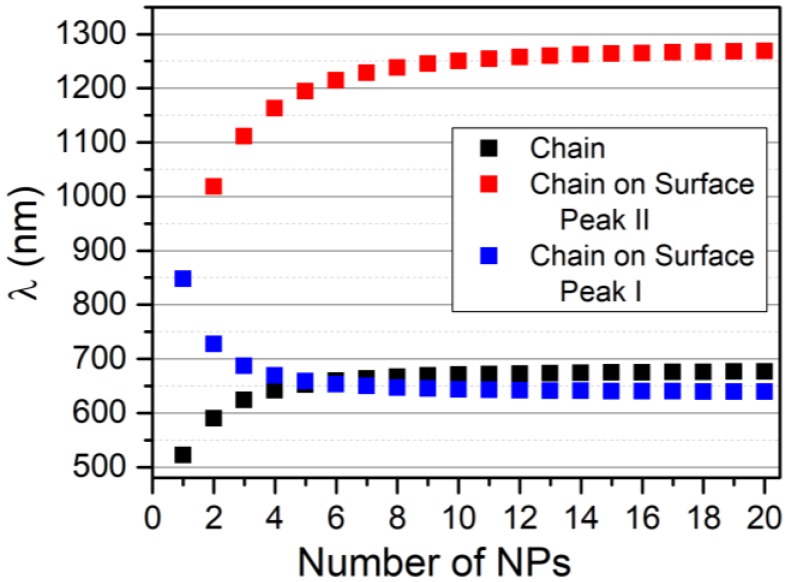
The lowest and highest energy bright mode of the chain on the surface (white square) and the chain (black square) as a function of the number of NPs in the chain. Values of ω_0_ and β taken from Reference [22] and the value of α = −2.6 eV.

Results are shown in Figure 10, which depicts the trend of Peak I and II, interpreted as the lowest and highest energy mode of the bright band, as a function of the chain length. It is observable that, while the group of modes of Peak II is slightly blue-shifted with respect to the free chain, Peak I is strongly red-shifted, which allows resonance until the near IR. The two peaks represent two different ways in which the metallic surface interacts with the chain: Peak I is obtained by a further stabilization of the system due to the localization of the metal plasmon with opposite charges with respect to the particle charge in contact with the surface. This stabilization of the charges of the entire system decreases the plasmonic energy. On the contrary, Peak II is the result of the stabilization of high energy modes of the chain due to the effect of the coupling of these modes with the surface plasmon of the metallic substrate. As the interaction of the dipole in the chain for these high energy modes increases, the energy of the system leads to an increasing number of elements in the chain. The resonance wavelength is slightly blue-shifted. 

**Figure 11:**
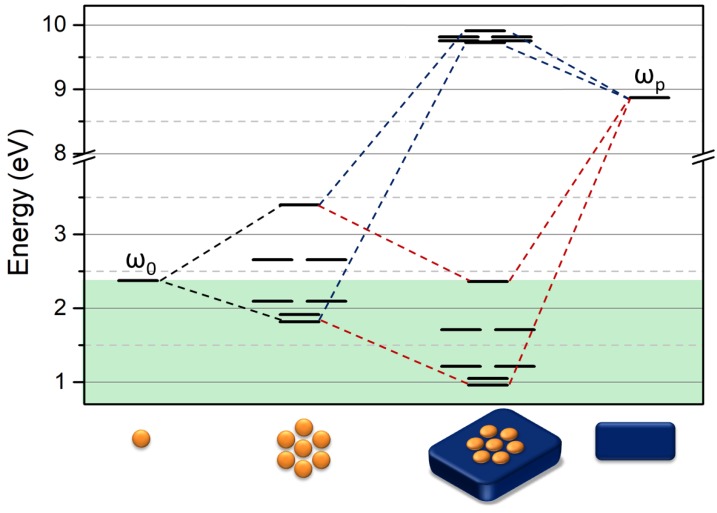
Sketch of the linear combination in the Variational Method for the heptamer on a gold surface. The black lines symbolize modes of three different systems (from left to right): heptamer cluster, heptamer cluster on the surface, and the surface with plasmon energy ω_p_ = 8.87 eV. The green region illustrates the position of bright mode ω < ω_0_ with ω_0_ = 2.38 eV.

The same approach can be applied to other geometries, as shown in Figure 11, for the case of the heptamer cluster. As a general result, the coupling of the NPs cluster with the surface induces an evident red-shift of the modes and, consequently, some of those levels that were dark modes become bright modes. This is a further enlargement of the resonance band and a new peak in the left side of the band. It is analogical to what has been discussed in case of the chain coupled to the metallic surface. If the heptamer is tested with the VM at the experimental conditions used for the chain system discussed previously, the maximum of the resonance peak is expected at 1380 nm. Moreover, a resonance mode at the same energy of the single NPs is formed as well as a narrow band of levels at a high energy, as already observed in the case of the chain.

## 6. Conclusions

In conclusion, the most important advantage of the proposed method is the rapid response and ease of application to all kinds of chain and cluster NPs systems, if the basic interactions of the elements of the system are known. This means that this approach is unable to determine the dependence of the energy shift due to the interparticle distance and particle size. However, it enables us to estimate how a given interaction changes as a result of an increasing number of conjugated NPs. This is possible without having to resort to a time-consuming calculation routine. As a consequence of a higher number of dipoles involved constructively in the state with lower energy, the corresponding wavelength represents the wavelength of the peak of the system’s resonance band. On the contrary, the whole band, with this quantum chemistry approach, is the result of the convolution of all the set of modes that are obtained by solving the secular determinant. The latter observation suggests that the incident electromagnetic radiation can polarize the NPs dipoles in a wide range of wavelengths.

Lastly, the aim of the application of this methodology is to support, by means of a rapid overview of the resonance wavelength characteristics of the system, the realization of more complex models as well as of experiments at nanoscale. Moreover, the present approach allows us to investigate the effect of the geometrical distribution of the NPs system, as well as the effect of substrates in an immediate way and this can be useful for selecting the laser source in laser-based analytical techniques as well as for selecting optimal substrates for the analysis. Although, in the present study, we focused our efforts on introducing a new model for interpreting the plasmon resonance in complex NPs system. In a future work, the use of the obtained wavefunctions will be investigated for estimating other properties of the plasmonic resonance from a more applicative point of view.

## Figures and Tables

**Table 1:** Dimer resonance energy from different published works for Ag and Au NPs, to be used in VM for the investigation of more complex system. In the table ω_0_, w_dimer_, Δω_dim_ correspond to single nanoparticle resonant energy, dimer resonant energy, and the energy shift, respectively.

NPs	Size (nm)	Id (nm)	Medium	ω_0_	ω_dimer_longitudinal mode_	|Δω|	Reference
AuNPs	20	0.5	Water	2.36	1.99	0.37	[21]
AuNPs	40	1	Air	2.37	2.09	0.28	[22]
AuNPs	18	3.8	water	2.36	2.32	0.04	[23]
AuNPs	18	0.2	Water	2.36	1.97	0.39	[23]
AuNPs	40	10	Water	2.34	2.34	0	[23]
AuNPs	10	0.8	Water	2.34	1.97	0.37	[23]
AuNPs	64	1	air	2.21	1.77	0.44	[24]
AuNPs	60	1	n = 1	2.05	1.88	0.17	[25]
AuNPs	60	1.5	n = 1.5	2.21	1.65	0.56	[25]
AuNPs	40	1.5	water	2.36	2.03	0.33	[26]
AuNPs	80	1	water	2.17	1.59	0.58	[27]
AuNPs	35	0.34	Vacuum (STEM)	2.34	2.09	0.25	[28]
AgNPs	60	3	Air	3.31	2.67	0.64	[29]
AgNPs	50	1	Air	3.10	2.58	0.52	[22]
AgNPs	36	2	Air	2.95	2.38	0.57	[30]
AgNPs	30	0.3	Vacuum (STEM)	2.91	2.45	0.46	[28]

**Table 2:** Energy and wavelength of the peak of the resonance band for different cluster geometries considering ω_0_ = 2.375 eV (522 nm) for single AuNP and β = −0.28 eV.

CLUSTER	ω (eV)	λ (nm)
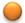	2.38	522
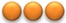	1.98	625
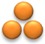	2.10	570
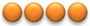	1.94	638
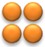	2.02	615
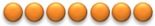	1.86	668
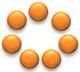	1.88	661
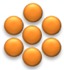	1.82	683
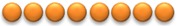	1.84	674
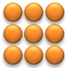	1.94	641
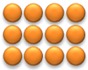	1.88	661
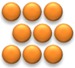	1.82	683

## Supplementary Materials

The following are available online at https://www.mdpi.com/2079-4991/9/7/929/s1; Figure S1: Examples of secular determinant of various NPs system geometry.
