# Supplementary material for: A Quantum Chemistry Approach Based on the Analogy with π-System in Polymers for a Rapid Estimation of the Resonance Wavelength of Nanoparticle Systems
Source: Nanomaterials (Basel). 2019 Jun 28;9(7):929. doi: 10.3390/nano9070929 (PMC6669735; doi:10.3390/nano9070929)
Supplement: Supplementary file 1 [file nanomaterials-09-00929-s001.pdf]

Supplementary Materials

$$\begin{vmatrix} \omega_0-\omega & \beta & \beta \\ \beta & \omega_0-\omega & \beta \\ \beta & \beta & \omega_0-\omega \end{vmatrix} = 0$$

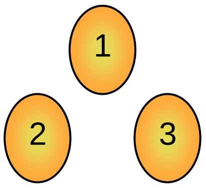

$$\begin{vmatrix}
\omega_0-\omega & \beta & 0 & \beta & \beta' & 0 & 0 & 0 & 0 \\
\beta & \omega_0-\omega & \beta & \beta' & \beta & \beta' & 0 & 0 & 0 \\
0 & \beta & \omega_0-\omega & 0 & \beta' & \beta & 0 & 0 & 0 \\
\beta & \beta' & 0 & \omega_0-\omega & \beta & 0 & \beta & \beta' & 0 \\
\beta' & \beta & \beta' & \beta & \omega_0-\omega & \beta & \beta' & \beta & \beta' \\
0 & \beta' & \beta & 0 & \beta & \omega_0-\omega & 0 & \beta' & \beta \\
0 & 0 & 0 & \beta & \beta' & 0 & \omega_0-\omega & \beta & 0 \\
0 & 0 & 0 & \beta' & \beta & \beta' & \beta & \omega_0-\omega & \beta \\
0 & 0 & 0 & 0 & \beta' & \beta & 0 & \beta & \omega_0-\omega
\end{vmatrix} = 0$$

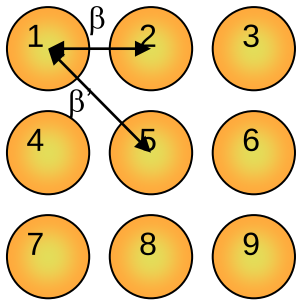

$$\begin{vmatrix}
\omega_0-\omega & \beta & 0 & \beta & 0 & 0 & 0 & 0 & 0 \\
\beta & \omega_0-\omega & \beta & \beta & \beta & 0 & 0 & 0 & 0 \\
0 & \beta & \omega_0-\omega & 0 & \beta & \beta & 0 & 0 & 0 \\
\beta & \beta & 0 & \omega_0-\omega & \beta & 0 & \beta & \beta & 0 \\
0 & \beta & \beta & \beta & \omega_0-\omega & \beta & 0 & \beta & \beta \\
0 & 0 & \beta & 0 & \beta & \omega_0-\omega & 0 & 0 & \beta \\
0 & 0 & 0 & \beta & 0 & 0 & \omega_0-\omega & \beta & 0 \\
0 & 0 & 0 & \beta & \beta & 0 & \beta & \omega_0-\omega & \beta \\
0 & 0 & 0 & 0 & \beta & \beta & 0 & \beta & \omega_0-\omega
\end{vmatrix} = 0$$

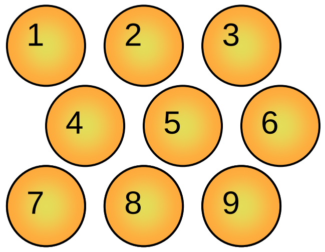

Figure S1. Examples of secular determinant of various NPs system geometry.
